# Molecular Subtype Classification Is a Determinant of Non-Sentinel Lymph Node Metastasis in Breast Cancer Patients with Positive Sentinel Lymph Nodes

**DOI:** 10.1371/journal.pone.0035881

**Published:** 2012-04-26

**Authors:** Wenbin Zhou, Zhongyuan He, Jialei Xue, Minghai Wang, Xiaoming Zha, Lijun Ling, Lin Chen, Shui Wang, Xiaoan Liu

**Affiliations:** Department of Breast Surgery, The First Affiliated Hospital with Nanjing Medical University, Nanjing, China; Health Canada, Canada

## Abstract

**Background:**

Previous studies suggested that the molecular subtypes were strongly associated with sentinel lymph node (SLN) status. The purpose of this study was to determine whether molecular subtype classification was associated with non-sentinel lymph nodes (NSLN) metastasis in patients with a positive SLN.

**Methodology and Principal Findings:**

Between January 2001 and March 2011, a total of 130 patients with a positive SLN were recruited. All these patients underwent a complete axillary lymph node dissection. The univariate and multivariate analyses of NSLN metastasis were performed. In univariate and multivariate analyses, large tumor size, macrometastasis and high tumor grade were all significant risk factors of NSLN metastasis in patients with a positive SLN. In univariate analysis, luminal B subgroup showed higher rate of NSLN metastasis than other subgroup (*P* = 0.027). When other variables were adjusted in multivariate analysis, the molecular subtype classification was a determinant of NSLN metastasis. Relative to triple negative subgroup, both luminal A (*P* = 0.047) and luminal B (*P* = 0.010) subgroups showed a higher risk of NSLN metastasis. Otherwise, HER2 over-expression subgroup did not have a higher risk than triple negative subgroup (*P* = 0.183). The area under the curve (AUC) value was 0.8095 for the Cambridge model. When molecular subtype classification was added to the Cambridge model, the AUC value was 0.8475.

**Conclusions:**

Except for other factors, molecular subtype classification was a determinant of NSLN metastasis in patients with a positive SLN. The predictive accuracy of mathematical models including molecular subtype should be determined in the future.

## Introduction

Sentinel lymph node biopsy (SLNB) has been proved to be a valid method of assessing axillary lymph node status in early breast cancer patients [1], [2], and has been accepted as a standard of care for early breast cancer patients [3], [4]. The axillary lymph node dissection (ALND) can be omitted when sentinel lymph nodes (SLNs) are negative. Generally, completion ALND is still needed for the patients with a positive SLN, and more morbidity would be carried including lymphoedema, seroma, arm weakness and so on [5], [6]. However, metastases in non-sentinel lymph nodes (NSLN) were found in about 40% of the patients with positive SLNs [7], [8].

Up to now, whether ALND is necessary for the patients with only SLNs involvement is not very clear. However, many researchers think that the therapeutic benefit is minimal for those patients [9]–[11]. Therefore, it is important to identify patients with SLNs involvement but without NSLNs metastases. Many clinical parameters were reported as risk factors of additional disease in NSLNs, including size of the primary tumor, size of the SLN metastasis, lymphovascular invasion, proportion of positive SLNs and so on [10], [12]. Furthermore, many mathematical models for estimation of NSLN metastases have been suggested in those patients [13]–[17]. However, the predicted probability of these models was not always very high.

The molecular subtype classification was firstly reported by Perou and his colleagues [18]. Different subtypes of breast cancer were associated with different metastasis pattern [19] and different survival [20]. The molecular subtype was associated with the axillary status. Recent studies [21], [22] showed that molecular subtypes based on immunohistochemical (IHC) were strongly associated with SLN status. To our knowledge, triple negative breast cancer (estrogen receptor (ER) negative, progesterone receptor (PR) negative, and HER2 negative) was correlated to more aggressive behaviors than other subgroups but with less lymph node metastases [21], [22]. According to these results, we hypothesized that the NSLN metastasis is correlated to intrinsic biological properties in different molecular subtypes regardless of the size of the primary tumor, grade of primary tumor, and size of the SLN metastasis.

The Cambridge model [14] was a modified predictive model for NSLN derived from the Memorial Sloan-Kettering Cancer Center (MSKCC) nomogram [13], which requires only three variables (tumor type/grade, maximum size of involved SLN, and proportion of positive SLNs). Previous studies [10], [23] suggested that the Cambridge model had the advantage of requiring fewer measurements with a more accurate predictive performance. The second aim of this study was to determine whether molecular subtype classification based on IHC can increase the predictive accuracy of the Cambridge model.

## Materials and Methods

### Patients

This retrospective study was approved by the ethics committee of The First Affiliated Hospital with Nanjing Medical University. Written consent was given by the patients for their information to be stored in the hospital database and used for research. This study was also in compliance with the Helsinki Declaration.

We reviewed our database of breast cancer patients who underwent SLNB from January 2001 through March 2011 in our hospital. Of these patients, 408 were identified who underwent complete ALND. In all, data from 130 women with a positive SLN who underwent ALND were included.

Medical records of all these 130 patients were reviewed by us. Clinical information collected for this study included age, tumor size, tumor grade, number of positive SLNs, number of negative SLNs, NSLN status, lymphovascular invasion, size of largest metastasis in the SLN, ER, PR, and HER2 status.

The SLNB procedure was performed with blue dye alone, or a combination with radioisotope. Preoperative SLN imaging was done on the day before surgery, according to a standard protocol, with radioisotope injected superficially. This was followed by γ camera imaging. On the day of surgery, the blue dye was injected, and the area was massaged. Most SLNs in this group were found along sentinel lymphatic channels (SLCs).

### Histology

After the SLNs were successfully dissected, they were sent to the pathology lab immediately. The metastases of SLNs were detected by imprint cytology, frozen section, hematoxylin and eosin (H&E) stain and IHC.

ALND was performed after SLN dissection when metastases were found in SLNs at intraoperative examinations. All NSLNs were analyzed with routine H&E stain on a single section of each node. When the definitive analysis revealed a positive SLN after the intraoperative examination, the patients were recalled for ALND in about two weeks. The H&E stain of all positive SLNs were reviewed by two experienced pathologists, and the maximum size of the metastasis was obtained. Positive SLNs were divided into two groups according to the maximum size of the metastasis: micrometastasis (≤2 mm) and macrometastasis (>2 mm).

ER and PR status were determined by IHC. The determination of HER2 over-expression status was determined according to the American Society of Clinical Oncology guidelines [24]. According to different combinations of ER, PR and HER2 status, all patients were categorized into four subgroups [25] as follows: luminal A, luminal B, HER2 over-expression, and triple negative.

### Statistical analysis

In our study, median, percentiles and range were analyzed for continuous variables. The candidate explanatory variables in the univariate and multivariate analyses of NSLN were: age at diagnosis, primary tumor size, tumor grade, number of SLN examined, maximum size of SLN metastasis, proportion of positive SLN, and molecular subtype classification. The patients were divided into two subgroups for univariate analyses: patients with NSLN metastasis group and without NSLN metastasis group. Differences between the two subgroups with regard to above variables were examined using Fisher's exact test for unordered categorical variables and nonparametric rank test for ordinal categorical variables. A multivariate logistic regression analysis was performed to determine the probability of having a positive NSLN and to build a nomogram. The variables for the Cambridge model in this study were: tumor grade, maximum size of involved SLN, and proportion of positive SLNs. The molecular subtype classification was added to the above three variables to build a new nomogram. Discrimination was quantified with the area under the receiver operating characteristic (ROC) curve. In all tests, a two-sided level of significance of 0.05 was applied. All data analyses were carried out using STATA version 10.0 and R software.

## Results

A total of 130 invasive breast cancer patients had a positive SLN and ALND. The median age of these patients was 50 y (range, 28–78 y). The average number of dissected SLNs was 2.4 (range, 1–7), and the median number of dissected NSLNs was 15 (range, 3–34). Of these 130 patients, seventy-six patients (58.46%) had at least one positive NSLN. Baseline characteristics of these 130 early breast cancer patients were shown in Table 1.

**Table 1:** Characteristics of breast cancer patients with positive sentinel lymph node.

Variable	No. (%)
**Age, y**	
≤50	71 (54.62%)
>50	59 (45.38%)
**Tumor size**	
T1	52 (40.00%)
T2	68 (52.31%)
T3	6 (4.62%)
NA	4 (3.08%)
**Tumor type**	
Ductal	124 (95.38%)
Others	6 (4.62%)
**Lymphovascular invasion**	
Yes	18 (13.85%)
Others*	112 (86.15%)
**Number of SLN exmanined**	
1	57 (43.85%)
2	36 (27.69%)
≥3	37 (28.46%)
**Size of SLN metastasis**	
Micrometastasis	32 (24.62%)
Macrometastasis	95 (73.08%)
NA	3 (2.31%)
**Grade**	
I	9 (6.92%)
II	38 (29.23%)
III	25 (19.23%)
NA	58 (44.62%)
**Molecular subtype**	
Triple negative	16 (12.31%)
Luminal A	69 (53.08%)
Luminal B	21 (16.15%)
HER2 over-expression	11 (8.46%)
NA	13 (10.00%)
**NSLN metastasis**	
Yes	76 (58.46%)
No	54 (41.54%)

* the data of most patients in this group was not available.

NSLN, non-sentinel lymph node; NA, not available.

In the univariate analysis of NSLN metastasis (Table 2), more large tumors were observed in positive NSLN group than in negative NSLN group (*P* = 0.046). The proportion of patients with macrometastasis in SLN was significantly different between the two groups (57.69% vs 86.67%, *P*<0.001). Furthermore, tumor grade between the two groups were significantly different (*P* = 0.003). The proportion of patients with metastases in all SLNs in positive NSLN group showed a trend towards higher than that in negative NSLN group (*P* = 0.081). The age at diagnosis (*P* = 1.00) and the number of SLN examined (*P* = 0.278) did not show significant differences between the two groups. Among molecular subtypes, luminal B subgroup showed the higher rate of NSLN metastasis than other groups (80.95% vs 53.13%, *P* = 0.027).

**Table 2:** Univariate analysis of NSLN metastasis.

Variable	Negative NSLN	Positive NSLN	*P* value
**Age, y**			
≤50	29	42	1.00
>50	25	34	
**Tumor size**			
T1	27	25	**0.046**
T2+T3	25	49	
**Number of SLN examined**			
1	28	29	0.278
2	11	25	
≥3	15	22	
**Size of SLN metastasis**			
Micrometastasis	22	10	**<0.001**
Macrometastasis	30	65	
**Grade**			
I	6	3	**0.003**
II	20	18	
III	5	20	
**Proportion of positive SLN**			
<1	21	18	0.081
1	33	58	
**Molecular subtype**			
Luminal B	4	17	**0.027** *
Luminal A	33	36	
HER2 over-expression	5	6	
Triple negative	7	9	

* *P* value for luminal B versus others (luminal A, HER2 over-expression and triple negative).

In the multivariate analysis (Table 3), large tumor size (*P* = 0.013), large size of SLN metastasis (*P* = 0.039), high tumor grade (*P* = 0.038), and high proportion of positive SLNs (*P* = 0.014) were all significant factors of NSLN metastasis. Furthermore, when other variables were adjusted, the molecular subtype classification was a determinant of NSLN metastasis. Relative to triple negative subgroup, both luminal A (*P* = 0.047) and luminal B (*P* = 0.010) subgroups showed a higher risk of NSLN metastasis in patients with a positive SLN. Otherwise, HER2 over-expression subgroup did not have a higher risk than triple negative subgroup (*P* = 0.183).

**Table 3:** Multivariate analysis of non-SLN metastasis.

Variable	OR	95% CI	*P* value
**Age**	3.63	0.67–19.78	0.136
**Tumor size**	9.35	1.59–55.08	**0.013**
**Number of SLN examined**	1.99	0.78–5.09	0.151
**Size of SLN metastasis**	9.21	1.12–75.62	**0.039**
**Grade**	4.89	1.09–21.84	**0.038**
**Proportion of positive SLN**	15.50	1.73–139.16	**0.014**
**Molecular subtype**			
**Triple negative**	1	Reference	
**Luminal A**	17.10	1.03–282.61	**0.047**
**Luminal B**	60.22	2.69–1350.03	**0.010**
**HER2 over-expression**	9.50	0.35–260.76	0.183

**Figure 1:**
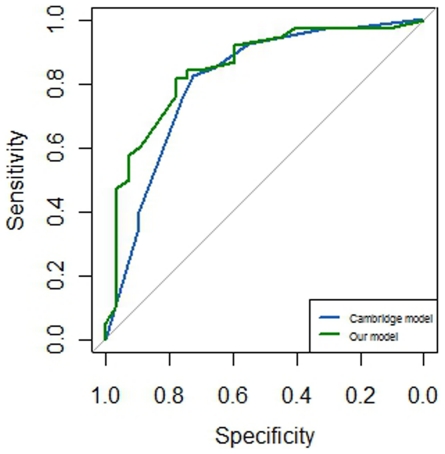
Receiver operating characteristic (ROC) curves of the Cambridge model and our model.

**Figure 2:**
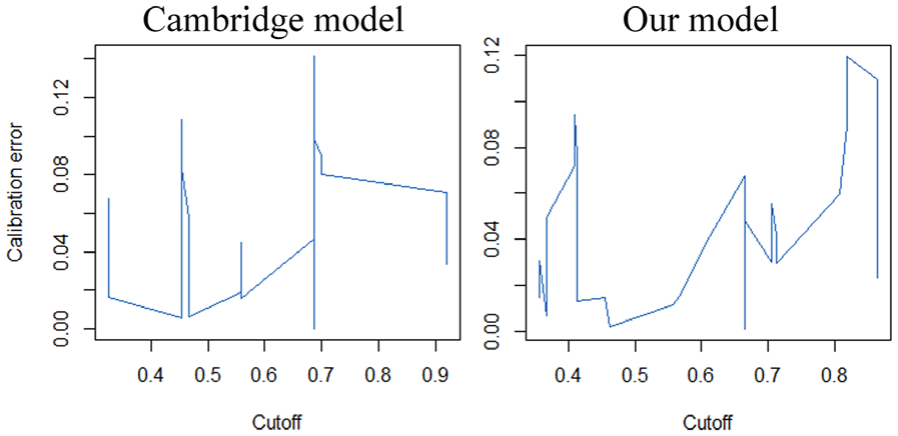
Calibration curves of the Cambridge model and our model.

Of all 130 patients, complete data from 69 patients were available for the Cambridge model. The discriminative ability of this model was shown in the ROC curve in Fig. 1. The value of the area under the curve (AUC) was 0.8095 (95% confidence interval (CI): 0.7011–0.9179). When the molecular subtype classification was added to the model, data from 65 patients were available for our new model. The AUC value was 0.8475 (95% CI: 0.7483–0.9466), which was higher than that of the Cambridge model. However, the 95% CIs overlapped, so no significant difference existed between the two curves. Furthermore, the relationship between the observed outcome frequencies and the predicted probabilities of the two models was shown in Fig. 2.

## Discussion

SLNB has been widely used to assess the axillary status for early breast cancer patients. However, there was no therapeutic purpose for patients with the only site of regional node disease in the SLN. Up to now, many risk factors for additional disease in NSLNs were identified [10], [12], including the size for primary tumor, the grade of primary tumor, the maximum size of positive SLNs, and lymphovascular invasion. Although ER, PR and HER2 status were analyzed in several studies [26]–[29], they were all not risk factors for NSLN metastasis in patients with a positive SLN.

Recent study [21] suggested that the molecular subtype classification was associated with SLN status. Compared with ER negative and HER2 negative subtype breast cancer, other subtypes showed a higher risk of SLN metastasis in multivariate logistic regression model. However, the relationship between the molecular subtype classification and NSLN status in patients with a positive SLN was still not clear. This study found that the molecular subtype classification was associated with NSLN metastasis in patients with a positive SLN. In our multivariate analysis, relative to triple negative breast cancer, both luminal A and Luminal B subtypes of breast cancer had a higher risk of NSLN metastasis; HER2 over-expression subtype breast cancer had a higher risk of NSLN metastasis, but no significant difference was reached. All these studies suggested that triple negative breast cancer may be associated with more aggressive behaviors but with less lymph node metastasis. Future studies with large sample size were still needed to validate our interesting findings.

Several models were created to predicting the probability of NSLN metastasis, including the MSKCC nomogram [13], the Mayo model [17], the Stanford Online Calculator model [15], the Cambridge model [14] and so on. The MSKCC model, which required nine variables, was validated in more than 15 studies [10], [30]. However, the AUC value from this model was not very high, ranging from 0.63 to 0.76. Previous studies [10], [30] suggested that the Cambridge model and Stanford model had the similar accurate predictive performance with the MSKCC and Mayo models, but required fewer measurements. A recent study [23] suggested that the Cambridge model had a highest AUC value out of all these models but required only three variables. Due to the advantages of the Cambridge model, it was selected for this study. The AUC value of the Cambridge model was 0.8095 in the present study and patient population, which seemed higher than that of previous studies. Small sample size in our study may contribute to this high AUC value of the Cambridge model. When the molecular subtype classification was added to the model, the AUC value was 0.8475 for this new model. Our new model may have a more accurate predictive performance, and future study with large sample size was needed to validate our finding.

### Limitations

On the other hand, some limitations still exist in the present study. First, most data of lymphovascular invasion, required in MSKCC model, was not available. Although the Cambridge model and our model did not require this variable, it should be considered in future studies. Second, the NSLNs were examined by routine histopathological analysis only, and the status of NSLNs may be underestimated. Third, the complete data used in Cambridge model and our model were from less than 70 patients. Therefore, future studies with large sample size should be taken.

In conclusion, in patients with a positive SLN, large tumor size, high tumor grade, macrometastasis in SLN, and high proportion of positive SLN were all independent predictors of NSLN involvement. When other variables were adjusted, the molecular subtype classification was associated with NSLN metastasis. Although our model had a more accurate predictive performance than the Cambridge model, no significant difference was found between the models in this study. Future prospective studies should be taken to validate our interesting findings.
